# Functional Characterization of Calcineurin Homologs *PsCNA1/PsCNB1* in *Puccinia striiformis* f. sp. *tritici* Using a Host-Induced RNAi System

**DOI:** 10.1371/journal.pone.0049262

**Published:** 2012-11-06

**Authors:** Hong Zhang, Jun Guo, Ralf T. Voegele, Jinshan Zhang, Yinghui Duan, Huaiyong Luo, Zhensheng Kang

**Affiliations:** 1 State Key Laboratory of Crop Stress Biology for Arid Areas and College of Plant Protection, Northwest A & F University, Yangling, Shaanxi, People's Republic of China; 2 Fachgebiet Phytopathologie, Institut für Phytomedizin, Fakultät Agrarwissenschaften, Universität Hohenheim, Stuttgart, Germany; 3 State Key Laboratory of Crop Stress Biology for Arid Areas and College of Plant Sciences, Northwest A & F University, Yangling, Shaanxi, People's Republic of China; Institute of Microbiology, Switzerland

## Abstract

Calcineurin plays a key role in morphogenesis, pathogenesis and drug resistance in most fungi. However, the function of calcineurin genes in *Puccinia striiformis* f. sp. *tritici* (*Pst*) is unclear. We identified and characterized the calcineurin genes *PsCNA1* and *PsCNB1* in *Pst.* Phylogenetic analyses indicate that *Ps*CNA1 and *Ps*CNB1 form a calcium/calmodulin regulated protein phosphatase belonging to the calcineurin heterodimers composed of subunits A and B. Quantitative RT-PCR analyses revealed that both *PsCNA1* and *PsCNB1* expression reached their maximum in the stage of haustorium formation, which is one day after inoculation. Using barely stripe mosaic virus (BSMV) as a transient expression vector in wheat, the expression of *PsCNA1* and *PsCNB1* in *Pst* was suppressed, leading to slower extension of fungal hyphae and reduced production of urediospores. The immune-suppressive drugs cyclosporin A and FK506 markedly reduced the germination rates of urediospores, and when germination did occur, more than two germtubes were produced. These results suggest that the calcineurin signaling pathway participates in stripe rust morphogenetic differentiation, especially the formation of haustoria during the early stage of infection and during the production of urediospores. Therefore *PsCNA1* and *PsCNB1* can be considered important pathogenicity genes involved in the wheat-*Pst* interaction.

## Introduction

Calcineurin, a serine-threonine-specific calcium/calmodulin-dependent protein phosphatase with two subunits (CNA and CNB), regulates a variety of physiological processes, such as growth, morphogenesis, pathogenicity, and membrane stress responses through the calcium signaling pathway in eukaryotes [1], [2]. The first fungal calcineurin genes were reported in 1991 from the budding yeast *Saccharomyces cerevisiae* [3], [4] and the filamentous fungus *Neurospora crassa* [5]. Many homologs of CNA or/and CNB have been found in medicinal fungi [6] and plant pathogens such as *Botrytis cinerea* [7] and *Magnaporthe oryzae* [8], [9]. Recent studies have confirmed that calcineurin controls virulence, hyphal elongation and multiple stress responses in the human pathogens *Candida dubliniensis* [10], *Cryptococcus neoformans* [11], [12], *Candida albicans* [13] and *Aspergillus fumigatus* [14], [15]. Similar findings have also been reported for the phytopathogens *Ustilago maydis* [16], *Cochliobolus miyabeanus* [17], and *Sclerotinia sclerotiorum* [18]. The calcineurin pathway also plays a role in drug resistance to azoles in *C. albicans* [19], [20], and in *C. dubliniensis* [10]. Inhibition of calcineurin can decrease fungal growth and arrest tissue invasion [21]. This opens possibilities to develop new antifungal agents targeting the calcineurin pathway in fungi [6].

RNA induced gene silencing or RNA interference (RNAi) is a complex natural phenomenon and a powerful reverse genetics tool for the analysis of gene function in eukaryotes [22]–[26]. In plants, virus-induced gene silencing (VIGS) was developed for rapid functional analysis of plant genes using viruses to deliver silencing constructs [27]–[29]. It has widely been applied in dicots such as *Arabidopsis* [30], tobacco [31] and tomato [32]–[33], and monocots such as barley [34] and wheat [35]–[38]. In fungi, RNAi technology has been deployed in more than 40 species including plant and human pathogens [39]. Nguyen et al. [40] developed a high-throughput RNA-silencing vector for *M. oryzae* to identify an involvement of calcineurin genes in colony pigmentation, sporulation, appressorium formation, and pathogenicity. However, for there is still no applicable transformation system available there are currently no techniques on hand for silencing genes in obligate biotrophic fungi directly. Host-induced gene silencing (HIGS) is a newly developed RNAi technology to indirectly silence parasite genes by expressing an RNAi construct *in vivo* in the host [41]. Host induced RNAi of three target genes suppressed their expression in the planthopper *Nilaparvata lugens* after feeding on rice plants [42]. Recent studies confirm the hypothesis that fungal genes can be suppressed *in planta* during interaction of the fungus with the host. Tinoco et al. [43] silenced the reporter gene GUS in *Fusarium verticillioides* by expressing *GUS* dsRNA in tobacco. HIGS was also successfully used in obligate biotrophic fungi. Using a BSMV-VIGS system expressing the target dsRNA in wheat, Nowara et al. [44] showed that the fungal genes *GTF1* and *GTF2* in *Blumeria graminis* play a role in haustorium formation and elongation of secondary hyphae. Yin et al. [45] also developed a BSMV-based HIGS approach to identify gene function in the biotrophic rust fungus *Puccinia striiformis* f. sp. *tritici* (*Pst*).

Wheat stripe rust, caused by the Basidiomycete *Pst*, is an important disease in wheat worldwide. As an obligate biotrophic pathogen infecting wheat leaves, *Pst* undergoes a high degree of morphological and physiological differentiation from urediospore to germ tube, invasive hypha and haustorium, a special structure for nutrient uptake from the host [46]–[48]. A few studies reported that the calcium signaling pathway is involved in the initial infection and biotrophic growth of rust fungi [49], [50]. Some homologs involved in calcium signaling such as CDPK were identified in *Pst* [49]. Recently, the PST-130 genome has been sequenced [51]. The sequences provided information to clone *Pst* genes involved in calcium signaling. In this study, we describe cloning, sequencing and transcription analysis of two calcineurin subunits from *Pst*, designated *PsCNA1* and *PsCNB1*. HIGS analysis using the BSMV-VIGS system and drug tests indicate a vital function in rust growth, development and sporulation.

## Results

### *PsCNA1* and *PsCNB1* encode calcineurin homologs

One of the expressed sequence tags (ESTs) from a full-length cDNA library of *Pst* [52] was found to be highly similar to PtCNA from *Puccinia triticina* (PTTG_07903) and PgCNA from *Puccinia graminis* f. sp. *tritici* (PGTG_14891). Another two ESTs from cDNA libraries contructed by Zhang et al. [49] and Ma et al. [53] are almost identical to the *CNB* genes from the other two wheat rusts (PTTG_02210 and PGTG_04308). Further sequencing of these clones from the Chinese *Pst* race CYR31, provided full-length cDNA sequences of *PsCNA1* and *PsCNB1* (Genbank accession numbers JX424819 and JX424820, respectively). The full length cDNA sequence for *PsCNA1* is 2,680 bp with an open reading frame (ORF) of 2,097 bp encoding a 698 amino acid (AA) protein, which consists of two calcineurin A domains and six Serine/threonine-protein phosphatase domains with a calculated molecular mass of 76.74 kDa (Fig. S1). The *PsCNB1* cDNA is 770 bp in length with an ORF of 528 bp encoding a 175 AA protein, with a molecular mass of 19.77 kDa, which has four calcium-binding EF-hand motifs and a N-myristoylation site (Fig. S2).

The levels of conservation of *Ps*CNA1 and *Ps*CNB1 are indicated in comparison with homologs from other fungi and some model organisms in Figure S1 and Figure S2, respectively. *Ps*CNA1 is 90% identical to the calcineurin A subunit of *P. triticina* and 84% identical to *P. graminis* f. sp. *tritici* CNA. *Ps*CNA1 is conserved among other fungi analyzed with 54% to 75%. *Ps*CNB1 exhibits strong similarity to calcineurin B proteins from other organisms. It is 100% identical to PtCNB, 99% identical to *Pg*CNB, and 60% to 83% identical to CNB genes from other fungi analyzed.

**Figure 1:**
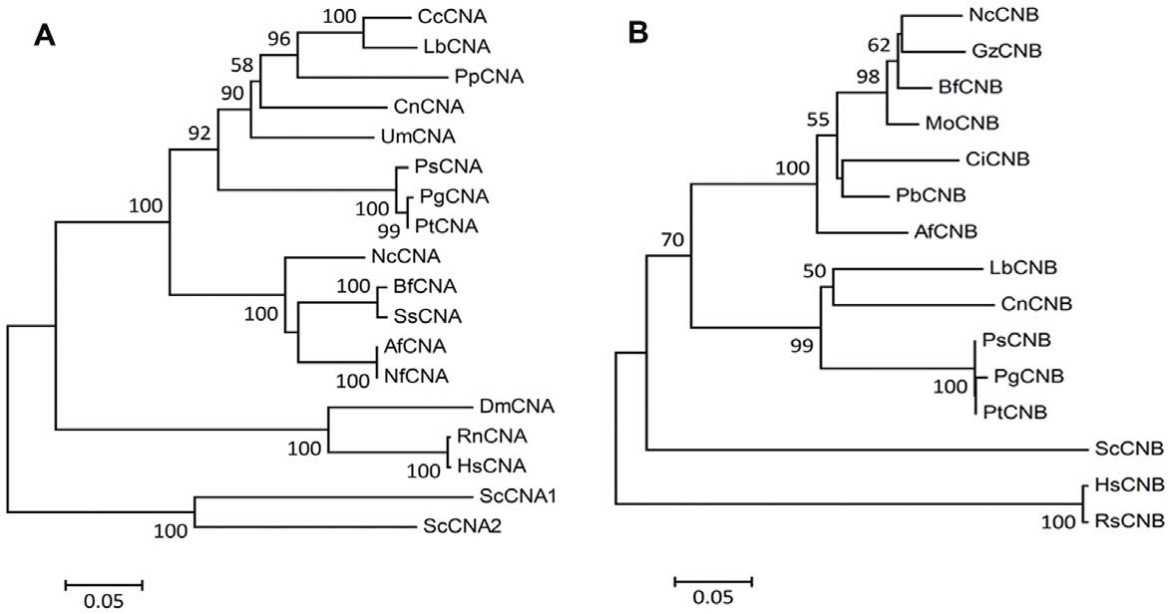
Phylogenetic analyses of CNA and CNB genes. A: *Af*CNA (*Aspergillus fumigatus*, XP_753703), *Bf*CNA (*Botryotinia fuckeliana*, XP_001558972), *Cc*CNA (*Coprinopsis cinerea*, XP_001838986), *Cn*CNA (*Cryptococcus neoformans* var. *grubii*, AAB97372), *Dm*CNA (*Drosophila melanogaster*, NP_727985), *Hs*CNA (*Homo sapiens*, NP_000936), *Lb*CNA (*Laccaria bicolor*, XP_001884713), *Nc*CNA (*Neurospora crassa*, XP_961193), *Nf*CNA (*Neosartorya fischeri*, XP_001259754), *Pg*CNA (*Puccinia graminis* f. sp. *tritici*, EFP89050), *Pp*CNA (*Postia placenta*, XP_002470453), *Ps*CNA (*Puccinia striiformis* f. sp. *tritici*, JX424819), *Pt*CNA (*Puccinia triticina*, PTTG_07903), *Rn*CNA (*Rattus norvegicus*, BAA14083), *Sc*CNA1 (*Saccharomyces cerevisiae*, SCRG_04371), *Sc*CNA2 (*Saccharomyces cerevisiae*, SCRG_01842), *Ss*CNA (*Sclerotinia sclerotiorum*, XP_001597594), *Um*CNA (*Ustilago maydis*, AAP48999). B: *Af*CNB (*Aspergillus flavus*, XP_002378292), *Bf*CNB (*Botryotinia fuckeliana*, XP_001555369), *Ci*CNB (*Coccidioides immitis*, XP_001248933), *Cn*CNB (*Cryptococcus neoformans* var. *neoformans*, XP_57033), *Gz*CNB (*Gibberella zeae*, XP_387580), *Hs*CNB (*Homo sapiens*, NP_000936), *Lb*CNB (*Laccaria bicolor*, XP_001884421), *Mo*CNB (*Magnaporthe oryzae*, ADD84607), *Nc*CNB (*Neurospora crassa*, CAA73345), *Pb*CNB (*Paracoccidioides brasiliensis*, XP_002795006), *Pg*CNB, (*Puccinia graminis tritici*, EFP78352), *Ps*CNB (*Puccinia striiformis* f. sp. *tritici*, JX424820), *Pt*CNB (*Puccinia triticina*, PTTG_02210), *Rs*CNB (*Rattus* sp., BAA03318), *Sc*CNB (*Saccharomyces cerevisiae*, SCRG_03838). The unrooted phylograms were constructed based on NJ analysis. Confidence of groupings was estimated by using 1,000 bootstrap replicates. Numbers next to the branching point indicate the percentage of replicates supporting each branch.

Phylogenetic analysis revealed that *Ps*CNA1 and *Ps*CNB1 cluster with other Basidiomycete fungi. Especially the three *Puccinia* sp. were most close to each other (Fig. 1). However, *Ps*CNA1 was closer to *P. graminis* f. sp. *tritici* than to *P. triticina* while *Ps*CNB1 was closer to *P. triticina* than to *P. graminis* f. sp. *tritici*.

### Suppressors block *Pst* germination

**Figure 2:**
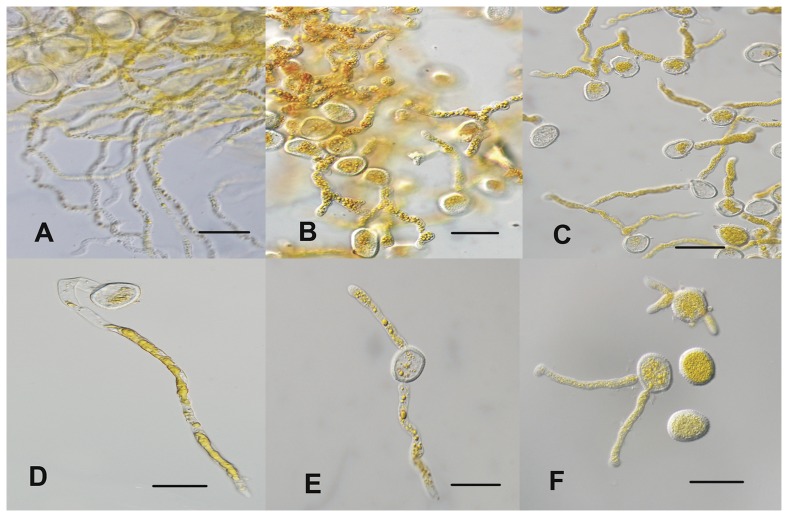
Immuno-suppressive drugs assay of *Pst* urediospores. *Pst* race CYR31 was treated with water (A, D), CsA (0.1 µM, B, E), or FK506 (1 µM, C, F). Treatment with either drug can limit the elongation of *Pst* germ tubes and block further differentiation. Analyses were performed using the light microscope; scale bar, 50 µm.

The immuno-suppressants cyclosporin A (CsA) and FK506 inhibit calcineurin activity and affect its function just like mutants of CNA or/and CNB in several fungi [6]. In order to test whether these two drugs affect the function of *PsCNA1* and *PsCNB1*, stripe rust urediospores were incubated with these drugs and germination was monitored. After 10 hours, microscopic analyses indicated that germination rate was reduced to 40.5% for FK506 (3 µM) and 66.5% for CsA (3 µM) treatment, compared to water (Table 1). Germ tubes of *Pst* were limited in their elongation by treatment with of FK506 (1 µM) or CsA (0.1 µM) compared to the control (Fig. 2C, 2B, 2A). Urediospores also frequently produced two or three more irregular germ tubes than the control (Fig. 2D, 2E, 2F).

**Table 1:** Germination rates of *Pst* (mean±SE).

Treatments	Percent Germination (10 hours)
Water	98.7±0.2^U^
FK506(3 µM)	40.5±4.7^U^
CsA (3 µM)	66.5±3.1^U^

U Values are significantly different at P = 0.05 according to the Tukey's test.

### Expression profiles of *PsCNA1* and *PsCNB1*

**Figure 3:**
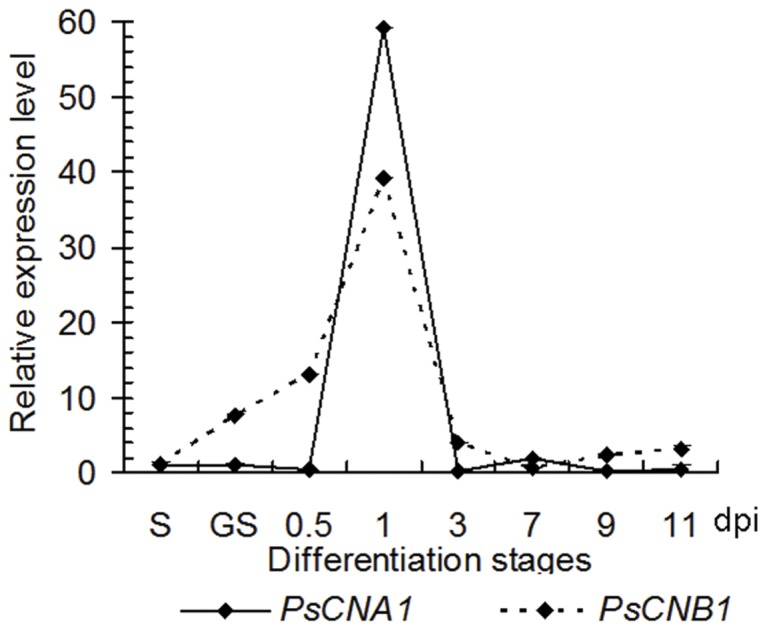
Transcript levels of *PsCNA1* and *PsCNB1* during *Pst* differentiation stages. RNA samples were isolated from leaves of wheat cultivar Suwon 11 inoculated with *Pst* race CYR31 at the indicated time points. Expression levels of *PsCNA1/PsCNB1* were estimated by the ΔΔCt method with the elongation factor gene of *Pst* as endogenous reference for normalization. S: urediospore, GS: germinated urediospore, dpi: days post inoculation.

To gain insight into the possible function of *Ps*CNA1 and *Ps*CNB1 in *Pst*, we investigated the expression of *PsCNA1* and *PsCNB1* (mRNA abundance) in different stages of *Pst* using quantitative PCR (qRT-PCR). *PsCNA1* and *PsCNB1* had similar expression profiles (Fig. 3). Transcript levels of both genes were drastically increased at 1.0 dpi, but quickly decreased to 3-fold, and 0.5-fold, respectively, at 11 dpi (Fig. 3). The maximum accumulation of transcript was 58 fold for *PsCNA1* and 38 fold for *PsCNB1*. However, kinetics of transcript accumulation differed between the two genes. The transcripts for *PsCNB1* drastically increased over time up to 1 dpi, whereas transcript level of *PsCNA1* only showed a dramatic increase at 1 dpi.

### HIGS for *PsCNA1* and *PsCNB1*

In order to determine the best inoculation day for *Pst* inoculation, virus symptoms were scored by visual assessment at four time point (8, 10, 12, 14 dpi) for the BSMV:γ:0-as vector (data not shown). Only three out of eighteen seedlings showed slight virus symptoms at 8 dpi. Symptoms increased at 10 dpi, and almost all seedlings showed 100% virus infection at 12 dpi. At 14 dpi leaves showed heavy symptoms with large yellow areas.Therefore, 12 dpi with BSMV was chosen for rust inoculation.

**Figure 4:**
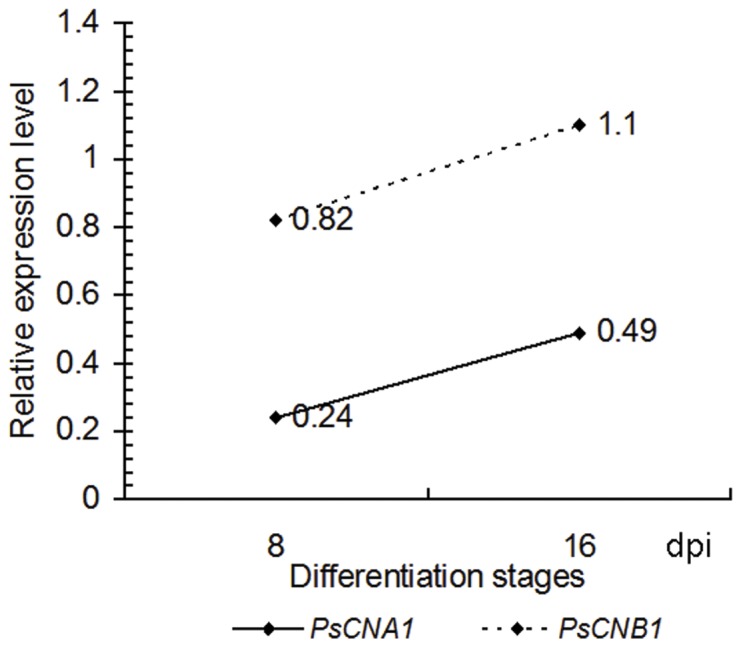
Transcript levels of *PsCNA1* and *PsCNB1* after HIGS during *Pst* differentiation stages. RNA samples were isolated from leaves infected with BSMV of wheat cultivar Suwon 11 inoculated with *Pst* race CYR31. Transcript levels of *PsCNA1* and *PsCNB1* were estimated by the comparative ΔΔCt method with elongation factor gene of *Pst* as the endogenous reference for normalization. dpi: days post inoculation.

In order to identify HIGS efficiency (knockdown rates), transcript levels of *PsCNA1* and *PsCNB1* were scored in inoculated silenced plants at 8 dpi by qRT-PCR. Results showed that silencing was detected for both BSMV vectors. *PsCNA1* transcript level exhibited an average of 24% expression in BSMV:γ:*PsCNA1*-as infected plants. However, *PsCNB1* transcript level showed only an average of 18% reduction. HIGS for both genes lost most effectiveness at 16 dpi (*PsCNA1*: 49% expression, *PsCNB1*: 110% expression) (Fig. 4).

### Silencing calcineurin blocks *Pst* growth and development in wheat leaves

**Figure 5:**
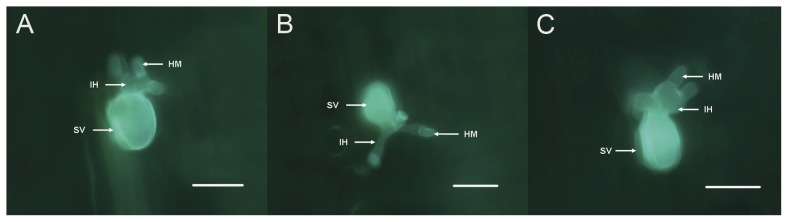
Histological observation of *Pst* growth using calcofluor staining. All wheat leaves were inoculated with *Pst* strain CYR31; fluorescence-microscopic analyses were done 2 days post inoculation. A: BSMV:γ:0-as (empty viral vector control); B: BSMV:γ:*PsCNA1*-as (silencing for *PsCNA1*); C: BSMV:γ:*PsCNB1*-as (silencing for *PsCNB1*); IH: infection hyphae; HM: haustorial mother cell; SV: substomatal vesicle. Scale bars: 50 µm.

To determine cytological changes associated with fungal growth on plants, silenced for *PsCNA1* or *PsCNB1* wheat leaves inoculated with race CYR31 were examined microscopically. Two time points (2 and 5 dpi) were compared. No significant differences in *Pst* development and hyphal growth were observed between control plants and plants carrying *PsCNA1* or *PsCNB1* knock-down constructs at 2 dpi (Table 2; Fig. 5A, 5B, 5C). However, at 5 dpi hyphal length on average in BSMV:γ:*PsCNA1*-as and BSMV:γ:*PsCNB1*-as infected wheat leaves were much shorter than those observed in controls (Table 2).

**Table 2:** Histological observation during HIGS (mean±SE).

Treatments^V^	Number of hyphal branches at 2 dpi^W^	Number of haustoria at 2 dpi^X^	Hyphal length at 5 dpi (µm)^Y^	Number of uredia at 16 dpi^Z^
BSMV:γ:0-as	2.00±0.28	3.21±0.32	283.94±10.41	99±7
BSMV:γ:*PsCNA1*-as	1.86±0.30	2.62±0.35	183.11±12.78	60±8
BSMV:γ:*PsCNb1*-as	1.82±0.17	2.59±0.17	197.41±13.09	68±5

Abbreviations: dpi, day post inoculation; SE, Standard Error.

V Leaves infected inoculated with BSMV:γ:0-as (empty vector), BSMV:*PsCNA*1-as and BSMV:*PsCNB*1-as followed by inoculation with CYR31.

Y Distance from the base of the substomatal vesicles to the hyphal tips.

W, X Values are not significantly different at P = 0.05 according to the Tukey's test.

Y, Z Values are significantly different at P = 0.05 according to the Tukey's test.

### Reduction in rust sporulation after silencing *Pst* calicineurin

**Figure 6:**
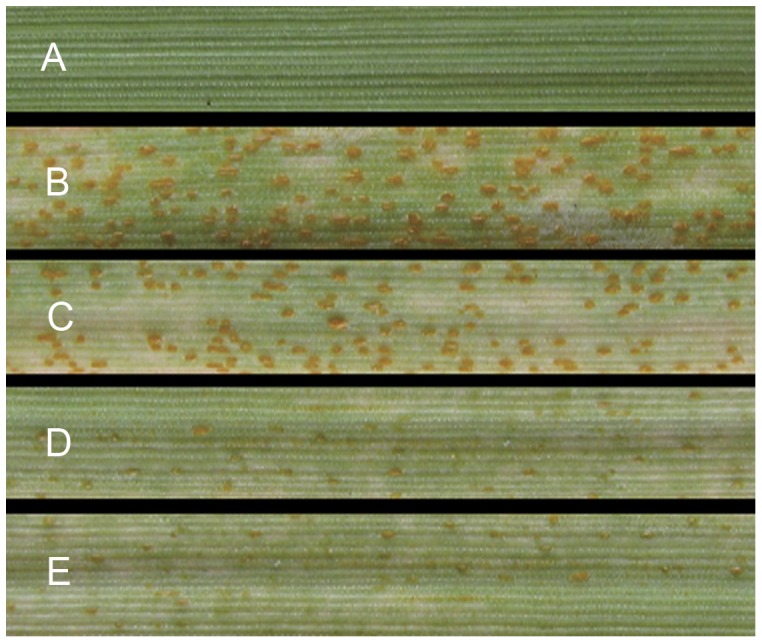
Uredia on silenced leaves 16 days after *Pst* inoculation. *Pst* development on wheat leaves after HIGS. A: No virus and Pst (healthy leaf control); B: No virus (normal infection with *Pst*); C: BSMV:γ:0-as (empty viral vector control); D: BSMV:γ:*PsCNA1*-as (*Pst* infection after silencing *PsCNA1*); E: BSMV:γ:*PsCNB1*-as (*Pst* infection after silencing *PsCNB1*).

**Figure 7:**
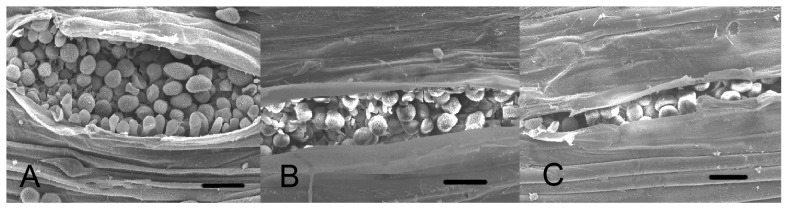
SEM photograph of uredia after HIGS at 16 dpi. Sorus of silenced *PsCNA1/PsCNB1* of *Pst* infected leaves inoculated with CYR31. Scanning electron micrographs by 600×, scale bar: 20 µm. A: BSMV:γ:0-as (empty viral vector control); B: BSMV:γ:*PsCNA1*-as (silencing *PsCNA1*); C: BSMV:γ:*PsCNB1*-as (silencing *PsCNB1*).

We also scored sporulation and found that the number of uredia was reduced on silencing plants (Fig. 6). Sporulation of *Pst* on silencing plants occurred two days later (12 dpi) than on the control plants (10 dpi). Statistical analyses 16 dpi determined an average number of 99 uredia for control plants, 60 uredia for BSMV:γ:*PsCNA1*-as, and 68 uredia for BSMV:γ:*PsCNB1*-as (Table 2). Uredia in silencing plants were smaller in size with open cavities that were shorter, and contained fewer spores (Fig. 7).

## Discussion

In this study we describe the isolation and characterization of two calcineurin genes from the wheat stripe rust fungus *Pst*. Phylogenetic analyses of eukaryotic *CNA* and *CNB* genes clearly show that *PsCNA1* and *PsCNB1* are closely related to calcineurin genes from other basidiomycetes. The calcineurin A/B protein family appears to be conserved in size and structure. Most *CNA* genes encode a protein of more than 500 amino acids and most CNB proteins contain about 175 amino acids [54]. All CNA proteins share high homology within the N-terminal 50 to 420 amino acid residues, but with significant variations in the C-terminal part. With a size of almost 700 aa *Ps*CNA1 is considerably larger than other CNA proteins (Fig. S1). In contrast, *Ps*CNB1 shares length, four conserved EF-hands, and a N-myristoylation site with other CNB proteins (Fig. S2). Especially the three *Puccinia* sp. have conserved amino acid residues at the N-myristoylation site and the first three EF motifs. Juvvadi et al. [15] found that CnaB is required for localization of CnaA to the septum and that the two calcineurin subunits are required to control hyphal growth and septation in *A. fumigatus*. *PsCNB1* is up-regulated earlier than *PsCNA1*, which might indicate that expression of *PsCNB1* is necessary for expression or stability of *PsCNA1*. Whether *Ps*CNB1 is indeed necessary for stabilization of *Ps*CNA1 and whether the two gene products co-operate still needs more research.

Harel et al. [18] reported that the transcript level of *CNA* in *S. sclerotiorum* is 2.5-fold higher in sclerotia than in infection hyphae. In contrast, our results from qRT-PCR show that transcript levels of *PsCNA1* and *PsCNB1* were much higher at 1 dpi, which corresponds to an early infection stage such as invasive hypha and initial haustorium formation. These differences in expression may be due to the fact that RNAs from *S. sclerotiorum* were prepared from mycelium grown on artificial medium, while RNAs of *Pst* were prepared from infected host plants (except germinated urediospores). Another reason may be different functions of calcineurin in the basidiomycete *Pst* and the ascomycete *S. sclerotiorum*.

Complexes of CsA with Cyclophilin A or/and FK506 with FK506-binding protein 12 (FKBP12) interfere with calcineurin binding to other phosphoprotein substrates in eukaryotes [1], [6]. The two inhibitors have been applied in numerous fungi to illustrate functions of calcineurin. CsA (10 µg/ml) induced cell-division patterns indistinguishable from mutants in calcineurin in *U. maydis* [16]. Both CsA and FK506 suppress growth of *C. neoformans in vitro* at 37°C [6], [11], and a similar phenomenon is seen in *C. dubliniensis* [10]. With FK506 and CsA treatment, *A. fumigatus* reveals stunted hyphae, more branches and defects of the conidiophore [55]. The immuno-suppressants CsA and FK506 led to germination defects in *Pst*. More than two or branched germ tubes appeared, which is a similar phenotypes as inhibitor treatment in *U. maydis* [16], or mutants of calcineurin in *Ustilago hordei* [56] or *A. fumigatus* [15].

Host induced gene silencing (HIGS) has succeeded to identify functions of parasite genes as an efficient reverse genetic tool in several fungi, insects and nematodes [41]. HIGS has emerged as parasite-derived resistance (PDR) to develop durable resistance in agricultural industry [41]. Virus induced gene silencing (VIGS) mediated by the barley stripe mosaic virus (BSMV) has been successfully developed in wheat [35], [36], and recently applied to HIGS with *B. graminis* [44] and *Pst* [45]. The BSMV-VIGS system is developed to express double stranded RNA (dsRNA) of targets from *Blumeria* and *Puccinia* genes in plants to trigger RNA silencing. We use BSMV-VIGS as viral vector to deliver *Pst* silencing constructs for calcineurin genes in order to silence them in *Pst* through the host. The suppression was almost 76% for *PsCNA1,* but only 18% for *PsCNB1* at 8 dpi (Fig. 4). Effective silencing was evident by the reduced number of uredia (Table 2. and Fig. 6), and smaller uredia with less open areas (Fig. 7). Our results illustrate different knock-down efficiencies for *Pst* HIGS by BSMV vectors with the two calcineurin genes. Silencing efficiency is variable for different genes or even the same gene. This has been shown for thirty-seven genes in *M. oryzae* [40], eleven genes of *Pst* [45], *inf1* in *Phytophthora infestans* [57] and *GUS* in *F. verticillioides* [43]. However, the phenotype of *PsCNB1* was similar to that of *PsCNA1* knock downs. An explanation might be that *PsCNA1* or/and *PsCNB1* act together to regulate rust sporulation. Less silencing of *PsCNB1* still led to an indistinguishable phenotype compared to the strong silencing of *PsCNA1.* This result might suggest that *PsCNA1* and *PsCNB1* are indispensable for each other because they have to compose a protein-complex for their function. *PsCNA1* and *PsCNB1* in *Pst* might join in the elongation or expansion of hyphae and sporulation.

Baulcombe [26] reported that plants have feedback mechanisms in RNA silencing. Fungi seem to exhibit a similar regulatory phenomenon. GUS expression and activity resumed to normal levels after seven generations in *F. verticillioides* [39]. Expressions of *gfp* and *inf1* are partially revcoverd in the Oomycete *P. infestans* [57]. Our results for *Pst* also showed recovery of expression of target genes after RNA silencing. The expression of silenced *PsCNA1*/*PsCNB1* genes increased up to almost normal expression levels during *Pst* development *in planta* (Fig. 4). This might be due to self-repair or self-protection mechanisms for an RNAi dynamic balance. In developing hyphae and haustoria increasing normal RNA might break the balance of RNA silencing. These phenomena must be considered as drawback of RNAi [39], [58].

**Figure 8:**
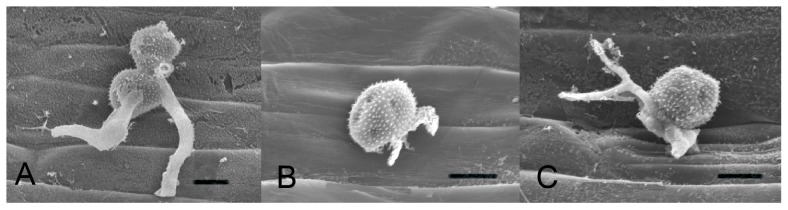
SEM photograph of germinated *Pst* urediospores after HIGS at 16 dpi. New urediospores of silenced *PsCNA1/PsCNB1* of *Pst* CYR31 in wheat leaves after 6 hour mist moisture. SEM micrographs for A: BSMV:γ:0-as (empty viral vector control), 1500×; B: BSMV:γ:*PsCNA1*-as (silencing *PsCNA1*), 2000×; C: BSMV:γ:*PsCNB1*-as (silencing *PsCNB1*), 2000×. Scale bars: 10 µm.

The silencing signal can be propagated to the offspring in plants and fungi. VIGS by BSMV vectors can be transferred to the next generation in wheat and barley [38]. Other VIGS vectors still also transmit silencing signals to next generation seedlings. Gene silencing by VIGS-ALSV (Apple Latent Spherical Virus) is from 33% of first progeny seedlings to 55% of subsequent progeny in soybean [27]. Silencing *gfp* was maintained in subsequent generations of *Moniliophthora perniciosa* [59]. We did not directly show BSMV heredity to next generation *Pst* urediospores. But after HIGS to *PsCNA1* and *PsCNB1,* we observed that the new urediospores also had similar defects with strange branched tubes (Fig. 8B, 8C) to that by the immuno-suppressors FK506 and CsA (Fig. 2E, 2F). These results illustrated that *PsCNA1* and *PsCNB1* take important function in the morphodifferentiation, hyphal development and sporulation in wheat stripe rust.

Although HIGS can be a valuable tool in identifying functions of fungal genes, the question is how the transfer of the silencing signals takes place. Fungal haustoria and the extrahaustorial matrix between host and fungus are specialized places to exchange nutrients and information [47], [48]. Nowara et al. [44] regarded the exosomal pathway as the means of secreted dsRNA or siRNA tranfer from the host plant wheat into *B. graminis*. By this way multivesicular bodies and exosomes transport RNAs to the extrhaustorial matrix. Yin et al [45] postulated that RNA silencing signals also extend from the expressing host cell into haustoria of *Pst.* Another possibility would be that the VIGS-BSMV vectors can cross the extrahaustorial matrix into haustoria in *Pst*. In this case the rust fungus needs a complete silencing machinery to accomplish degradation of RNAs. Although Argonaute-like proteins (AGO) have been identified in *Pst* (GenBank accession: AEM61140.1) [51], future work could answer these questions.

## Materials and Methods

### Strains and RNA isolation

Chinese *Pst* race CYR31 was inoculated and propagated on wheat cultivar Suwon 11 as described previously [53]. For isolating RNA from infected plants, infected wheat leaves were harvested at 0, 0.5, 1, 3, 7, 9 and 11 days post inoculation (dpi). All material was frozen in liquid nitrogen and stored at −80°C.

Total RNA was extracted using the Qiagen Plant RNeasy kit (Qiagen, Hilden, Germany) from sampled urediospores, germinated urediospores, and infected wheat leaves. First-strand cDNA was synthesized from 1 µg total RNA of each sample using the SMART™ reverse transcription Kit (Clontech Laboratories, Inc., Mountain View, CA) according to the manufacturer's instructions.

### Isolation and sequence analysis of *PsCNA1* and *PsCNB1*

Screening the *Pst* cDNA library constructed by Ling et al. [52], one EST clone (480 bp) was found to be homologous to CNA genes. Another two EST clones (zyh1090 and mjb959; GenBank accession: ES322265 and GR305110, respectively) with homology to CNB genes were obtained from a *Pst* cDNA library constructed by Zhang et al. [49] and a cDNA library from wheat leaves inoculated with *Pst* constructed by Ma et al. [53]. Two primers PsCNA-S/AS and PsCNB-S/AS (Table 3) were designed to get the full length cDNA sequence of *PsCNA1* and *PsCNB1* from *Pst*. The clones of *PsCNA1* and *PsCNB1* were sequenced on an ABI PRISM 3130XL Genetic Analyzer (Applied Biosystems, Carlsbad CA). Sequences were analyzed using NCBI BLAST (http://blast.ncbi.nlm.nih.gov/Blast.cgi), BLAST searches against the database of *P. graminis* f. sp. *tritici* and *P. triticina* (http://www.broadinstitute.org/annotation/genome/puccinia_group/GenomesIndex.html), and ORF Finder (http://www.ncbi.nlm.nih.gov/gorf/gorf.html). The alignments of the deduced protein sequences and phylogenetic trees were computed using MEGA4 and ClustalX version 1.83 as described by Guo et al. [60]. *PsCNA1* and *PsCNB1* sequences have been deposited in GenBank (GenBank accession number JX424819 and JX424820, respectively).

**Table 3:** Primers used in this study.

Primers	Sequence (5′ to 3′)
PsCNA-S	ATGTCGACCGGCCTACCCGAAT
PsCNA-AS	TTAAGATGCAGAACCAGAACCAGATG
PsCNB-S	ATGGGTCAAACCGGATCACAAC
PsCNB-AS	CTAAAATAGGGCCTCGAGTGTC
PsCNA-1VS	ATATTAATTAACAGCAGACGCAGGAGAA
PsCNA-1VAS	TATGCGGCCGCCTGGACTAGTAGGTACGG
PsCNB-1VS	ATATTAATTAAATGAAGTTGGATAGGGAC
PsCNB-1VAS	TATGCGGCCGCCCTCGACTGCTGAATGC
PsCNA-1RTS	CCTCAAAGGCGTCAACCGT
PsCNA-1RTAS	CGAGCAACCTCTGACGTGG
PsCNB-1RTS	TTGATTACCAGTCACGAGCAG
PsCNB-1RTAS	CGCTATCGCTGGAATCTGTA
PsCNB-2RTS	TGATGAAGATGGAGGAGGAAC
PsCNB-2RTAS	GTCTTATCAACGATCTGTTGGAGT
PsActin-1RTS	TTGGATTCTGGAGATGGTGTC
PsActin-1RTAS	CTCTTCGGCGGTGGTAGTGA
PsEF-1RTS	TTCGCCGTCCGTGATATGAGACAA
PsEF-1RTAS	ATGCGTATCATGGTGGTGGAGTGA

### Quantitative RT-PCR

To analyze the transcript levels of the two subunits of *Pst* calcineurin, relative quantification of gene expression was performed using quantitative RT-PCR (qRT-PCR) on an ABI prism 7500 sequence detection system (Applied Biosystems, Carlsbad, CA). Transcript abundance was assessed with three independent biological replicates. Amplification was performed as follows: 95°C for 1 min, followed by 40 cycles of 10 s at 95°C, 20 s at 60°C and 40 s at 72°C. This was followed by melting curve analysis. The transcript levels of *PsCNA1* and *PsCNB1* were calculated by the 2^−ΔΔCT^ method with the *EF1* gene of *Pst* as endogenous reference for normalization as described by Guo et al. [60]. The following primers were used for qRT-PCR *PsCNA1* (PsCNA-1RT S/AS, Table 3), *PsCNB*1 (PsCNB-1RT S/AS, Table 3). Relative quantification of *PsCNA1* and *PsCNB1* was computed for the different stages in comparison to that at zero hour incolulation with *Pst* urediospores.

### Construction of BSMV-based VIGS vectors and VIGS assay

BSMV-VIGS vectors are based on the constructs by Holzberg et al. [35]. To avoid non-specific silencing of wheat genes, the target regions of VIGS vectors were blasted for homologs to all wheat sequences in the NCBI database and designed to be rust-specific. Selected *PsCNA1* and *PsCNB1* gene fragments were amplified by PCR from *Pst* cDNA using primers with restriction enzymes *NotI* and *PacI* sites (Primers: PsCNA-1VS/AS and PsCNB-1VS/AS, respectively; Table 3). Amplicons were ligated into the BSMV γ vector generating BSMV:γ:*PsCNA1*-as and BSMV:γ:*PsCNB1*-as. The native BSMV:γ:0-as was used as negative control.

Two-leaf wheat seedlings were used for virus inoculation by rubbing the first leaf as described by Yin et al [45]. Seedlings were incubated in the greenhouse after spraying with water (25°C for 16 hours light and 20°C for 8 hours dark). After inoculation with *Pst* urediospores, plants were incubated at 20°C for 16 hours light and 16°C for 8 hours dark. Primers (PsCNA-1RT S/AS and PsCNB-2RT S/AS, respectively; Table 3) were used for assaying the transcript levels of *PsCNA1* and *PsCNB1*. Control seedlings were infected with the BSMV:γ:0-as vector and also inoculated with *Pst*. Total RNA was extracted from leaves of 18 wheat seedlings at two time points (8 and 16 days) after rust inoculation.

### Histological obervtion of *Pst* growth in wheat leaves

Wheat leaves were sampled at 2 and 5 days and stained with Calcofluor White. The infected leaves were examined with the microscope (Olympus BX-51) to observe *Pst* haustoria and infection hyphae under UV light. Wheat leaves infected *Pst* (16 dpi) were observed by scanning electron microscopy (JEM1230).

### Inhibitor assays using CsA or FK506

CsA (Sigma, USA) was diluted to be 1 mM mother solution in 95% ethyl alcohol. FK506 (Sigma, USA) was diluted to be 1 mM mother solution in DMSO. CsA and FK506 were added to 5 ml of sterile water with *Pst* urediospores to reach final concentrations of 3 µM and 0.1 µM for CsA, or 3 µM and 1 µM for FK506. Spore suspensions were incubated at 4°C for germination with sterile water-incubated urediospores as control. Germination was examined under the microscope after 10 hours.

## Supporting Information

Figure S1 **Comparison of** ***Ps*** **CNA1 to other homologous CNA proteins.** *Cn*CNA (*Cryptococcus neoformans* var. *grubii*, AAB97372), *Pg*CNA, (*Puccinia graminis tritici*, EFP89050), *Ps*CNB (*Puccinia striiformis* f. sp. *tritici*, JX424819), *Pt*CNA (*Puccinia triticina*, PTTG_07903), *Ss*CNA (*Sclerotinia sclerotiorum*, XP_001597594), *Um*CNA (*Ustilago maydis*, AAP48999). The solid arrow lines show the STPHPHTASE (Serine/threonine-protein phosphatase domains) domains and the dashed line shows the Cacineurin A Domain. Shaded regions show the same AA.
(TIF)

Figure S2 **Comparison of** ***Ps*** **CNB1 to other homologous CNB proteins.** *Bf*CNB (*Botryotinia fuckeliana*, XP_001555369), *Cn*CNB (*Cryptococcus neoformans* var. *neoformans*, XP_57033), *Mo*CNB (*Magnaporthe oryzae*, ADD84607), *Nc*CNB (*Neurospora crassa*, CAA73345), *Pg*CNB, (*Puccinia graminis tritici*, EFP78352), *Ps*CNB (*Puccinia striiformis* f. sp. *tritici*, JX424820), *Pt*CNB (*Puccinia triticina*, PTTG_02210), *Sc*CNB (*Saccharomyces cerevisiae*, SCRG_03838). The first solid arrow line show Myristrylation site, the other solid arrow lines show the EF-hands motifs. Shaded regions indicate the same AA.
(TIF)
