# Supplementary figures and images for: Functional Characterization of Calcineurin Homologs PsCNA1/PsCNB1 in Puccinia striiformis f. sp. tritici Using a Host-Induced RNAi System
Source: PLoS One. 2012 Nov 6;7(11):e49262. doi: 10.1371/journal.pone.0049262 (PMC3490909; doi:10.1371/journal.pone.0049262)

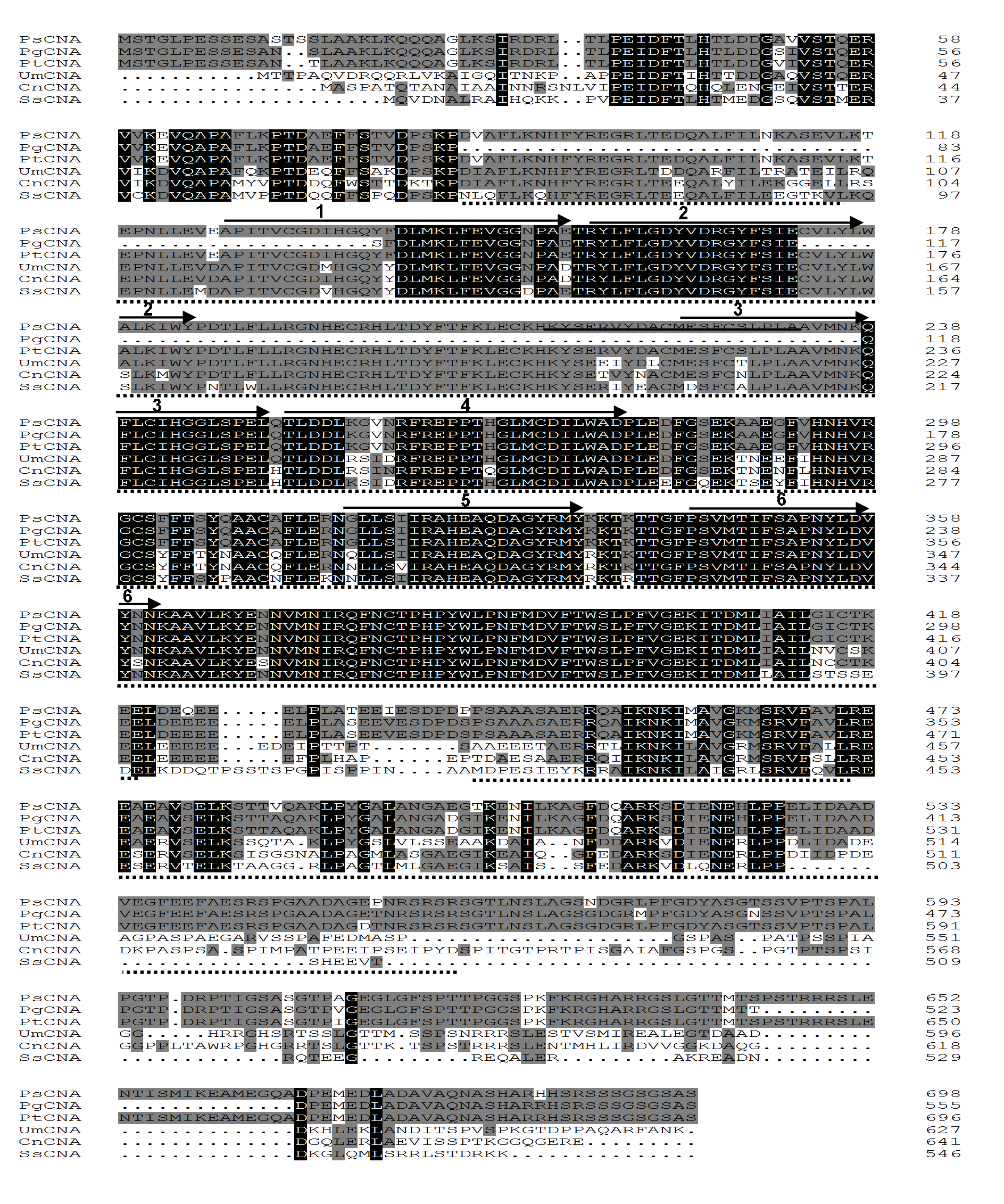

Supplement: Figure S1 — Comparison of Ps CNA1 to other homologous CNA proteins. CnCNA (Cryptococcus neoformans var. grubii, AAB97372), PgCNA, (Puccinia graminis tritici, EFP89050), PsCNB (Puccinia striiformis f. sp. tritici, JX424819), PtCNA (Puccinia triticina, PTTG_07903), SsCNA (Sclerotinia sclerotiorum, XP_001597594), UmCNA (Ustilago maydis, AAP48999). The solid arrow lines show the STPHPHTASE (Serine/threonine-protein phosphatase domains) domains and the dashed line shows the Cacineurin A Domain. Shaded regions show the same AA. (TIF) [file pone.0049262.s001.tif]

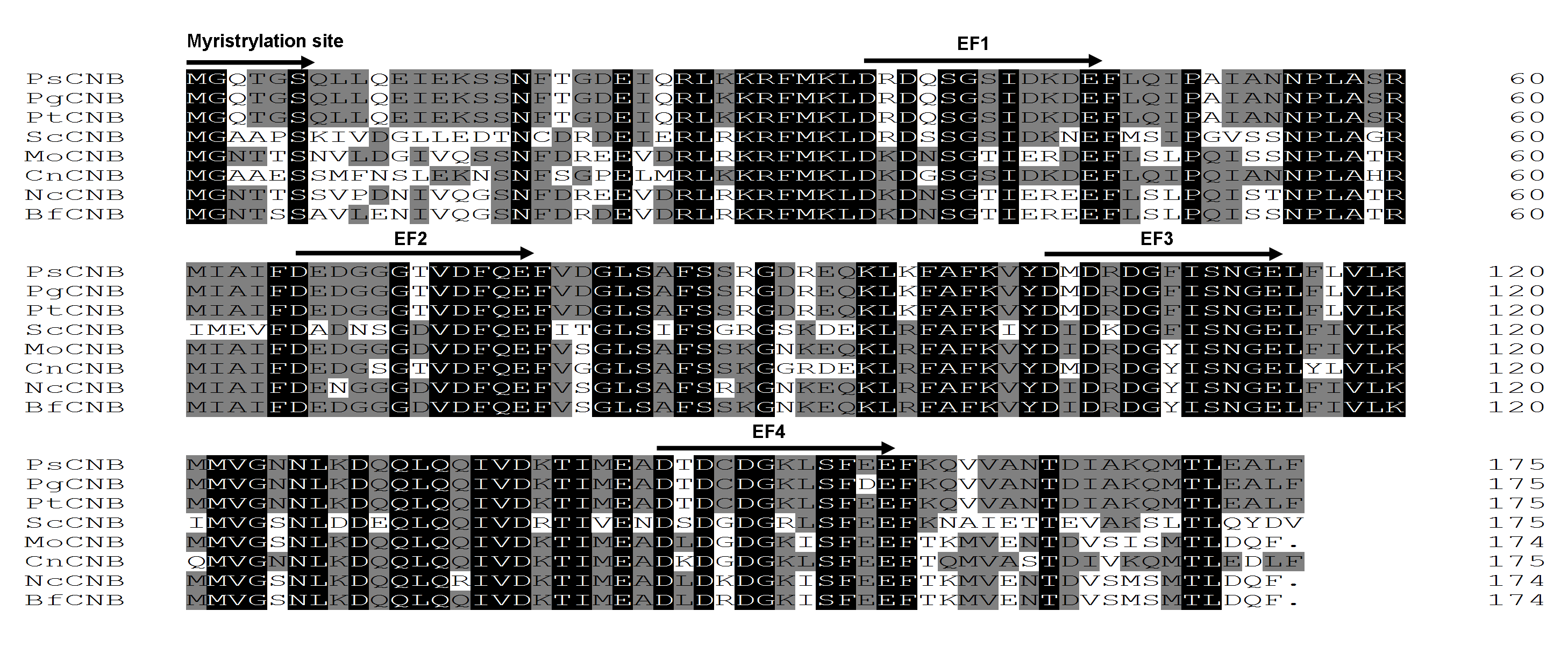

Supplement: Figure S2 — Comparison of Ps CNB1 to other homologous CNB proteins. BfCNB (Botryotinia fuckeliana, XP_001555369), CnCNB (Cryptococcus neoformans var. neoformans, XP_57033), MoCNB (Magnaporthe oryzae, ADD84607), NcCNB (Neurospora crassa, CAA73345), PgCNB, (Puccinia graminis tritici, EFP78352), PsCNB (Puccinia striiformis f. sp. tritici, JX424820), PtCNB (Puccinia triticina, PTTG_02210), ScCNB (Saccharomyces cerevisiae, SCRG_03838). The first solid arrow line show Myristrylation site, the other solid arrow lines show the EF-hands motifs. Shaded regions indicate the same AA. (TIF) [file pone.0049262.s002.tif]
